# FDA-cleared home sleep apnea testing devices

**DOI:** 10.1038/s41746-024-01112-w

**Published:** 2024-05-13

**Authors:** Ji Hyeun Park, Changwon Wang, Hangsik Shin

**Affiliations:** 1 Department of Convergence Medicine, Asan Medical Center, Brain Korea 21 Project, University of Ulsan College of Medicine, Seoul, 05505 Republic of Korea; 2 Biomedical Engineering Research Center, Asan Medical Center, Seoul, 05505 Republic of Korea

**Keywords:** Diagnosis, Sleep disorders

## Abstract

The demand for home sleep apnea testing (HSAT) devices is escalating, particularly in the context of the coronavirus 2019 (COVID-19) pandemic. The absence of standardized development and verification procedures poses a significant challenge. This study meticulously analyzed the approval process characteristics of HSAT devices by the U.S. Food and Drug Administration (FDA) from September 1, 2003, to September 1, 2023, with a primary focus on ensuring safety and clinical effectiveness. We examined 58 reports out of 1046 that underwent FDA clearance via the 510(k) and de novo pathways. A substantial surge in certifications after the 2022 pandemic was observed. Type-3 devices dominated, signifying a growing trend for both home and clinical use. Key measurement items included respiration and sleep analysis, with the apnea–hypopnea index (AHI) and sleep stage emerging as pivotal indicators. The majority of FDA-cleared HSAT devices adhered to electrical safety and biocompatibility standards. Critical considerations encompass performance and function testing, usability, and cybersecurity. This study emphasized the nearly indispensable role of clinical trials in ensuring the clinical effectiveness of HSAT devices. Future studies should propose guidances that specify stringent requirements, robust clinical trial designs, and comprehensive performance criteria to guarantee the minimum safety and clinical effectiveness of HSATs.

## Introduction

A sleep disorder is a condition that disrupts the normal sleep pattern, affecting the quality, timing, and duration of sleep and leading to difficulties in falling and staying asleep or achieving restorative sleep. Sleep apnea is a common sleep disorder that affects nearly one billion adults worldwide and has been linked to conditions such as daytime sleepiness, cardiovascular diseases, type-2 diabetes, fatty liver, and mood disorders^1–8^. A common method of measuring and analyzing sleep disorders is polysomnography (PSG). PSG measures various physiological signals, including the electroencephalogram (EEG), electrooculogram (EOG), electromyogram (EMG), respiration, and electrocardiogram (ECG) during sleep, allowing for complex monitoring of various body activities. However, PSG is limited to specialized medical facilities because it should be performed in a specific room and under the supervision of a professional facilitator; furthermore, it is limited to routine applications because it requires a long time to perform and is expensive^9,10^.

The home sleep apnea test (HSAT) allows patients to self-monitor their sleep without visiting a professional healthcare provider^11^. HSATs have the potential to improve access to sleep apnea diagnosis and patient convenience and reduce health care costs through at-home self-monitoring. Clinical studies of HSATs have reported that HSATs can effectively monitor sleep apnea and assist in higher-quality care^12,13^. Demand for at-home sleep testing and HSATs has increased since the coronavirus 2019 (COVID-19) pandemic^14^. However, whether HSATs can replace in-lab PSG remains a topic of controversy owing to concerns about their performance^15^; in particular, they often do not measure EEG, EOG, or chin EMG, causing difficulty in accurately measuring sleep stages and cortical arousal^16,17^. A recent meta-analysis of HSAT accuracy showed that the apnea–hypopnea index (AHI) assessment with HSAT has a significant number of false negatives and false positives, with significant interproduct variability^18^. They reported that the inadequate sensitivity and specificity render it an unsuitable alternative to PSG for detecting sleep apnea patterns for AHI ≥ 5, 15, and 30, suggesting that HSATs have limited potential for widespread use as an accurate clinical diagnostic tool at home. This may be because the accuracy of the test device varies depending on which version of the (constantly) revised American Society of Sleep Medicine (AASM) hypopnea criteria the reference device adopts^19^, or that inter-rater variability in sleep test results can occur even when performed in a laboratory^20,21^. However, all of these reasons ultimately refer to the standards and guidances used to evaluate HSAT devices and their clinical performances.

We aimed to examine the evolution of HSATs and explore the requirements for safer and more effective use of HSATs. We analyzed the United States (U.S.) Food and Drug Administration (FDA)-cleared HSAT devices and identified trends in the development of HSATs. We evaluated the mechanisms by which manufacturers have ensured safety and clinical effectiveness and suggested future directions for HSAT development.

## Results

### Search results

**Fig. 1:**
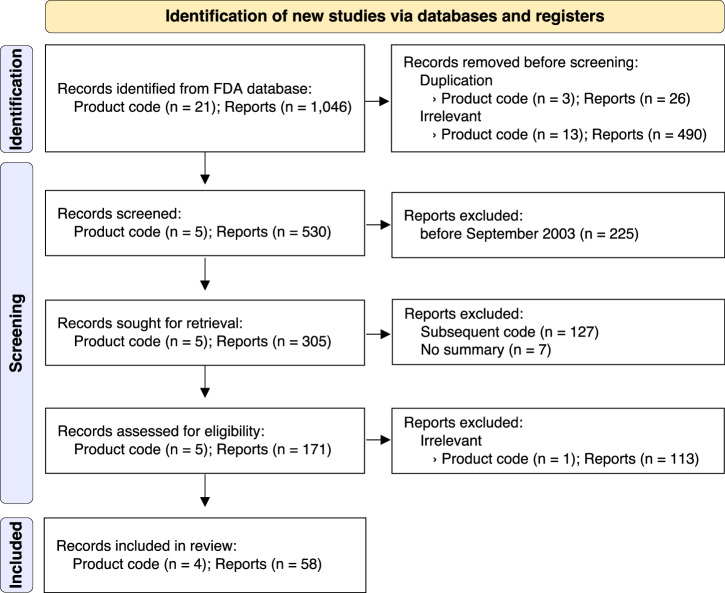
PRISMA 2020 flow diagram for the searching and screening reports of FDA-cleared HSAT products. FDA Food and Drug Administration, HSAT home sleep apnea testing, PRISMA preferred reporting items for systematic reviews and meta-analyses.

The data processing steps and results are shown in the Preferred Reporting Items for Systematic Reviews and Meta-Analyses (PRISMA) 2020 flow diagram (see Fig. 1). In total, 1046 reports with 21 product codes were retrieved using the search string, and 516 reports with 16 product codes were excluded as duplicates or determined to be irrelevant based on the initial screening process. At the screening stage, 225 reports with FDA clearance dates before September 2003, 127 reports with subsequence codes (rather than classification product codes), seven reports with missing summaries, and 113 reports unrelated to HSAT were sequentially excluded. Finally, 58 reports for four product codes (MNR, QRS, OLV, and OMC) were selected for analysis.

### Overview of FDA-cleared HSAT devices

**Fig. 2:**
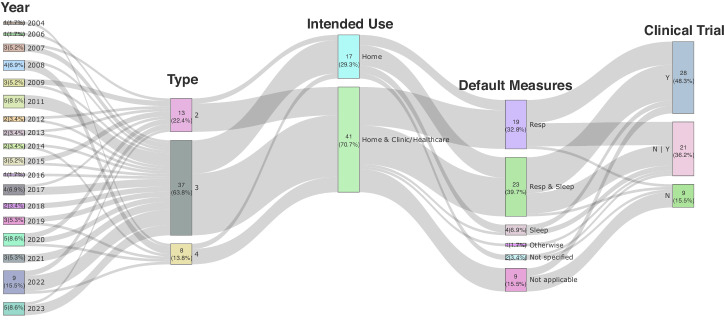
Sankey diagram for the overall trend of FDA-cleared HSAT devices. FDA Food and Drug Administration, HSAT home sleep apnea testing. N|Y: cases cleared the FDA by adding or changing features unrelated to efficacy to a product already cleared through clinical trials.

The overall trend for FDA-cleared HSAT devices is illustrated in Fig. 2. HSAT devices have consistently been obtaining FDA clearance each year and experienced a significant spike in 2022, during which nine (15.5%) products received clearance. Based on the traditional portable monitoring device classification^22^, we found that Type-3 devices comprise the largest percentage distribution of sleep monitoring devices (37, 63.8%), followed by Type-2 (13, 22.4%) and Type-4 (8, 13.8%) devices. A noticeable increase in the number of clearances of Type-3 devices over time was observed in the trend of sleep monitoring device types by the year of clearance, contrasting with the previous even distribution across all types. Conversely, Type-4 devices exhibit a decreasing trend over time. Regarding the distribution of intended use, 17 (29.3%) devices were designated for home use only, whereas 41 (70.7%) devices were universal devices capable of being used in home and clinic/healthcare environments. Most HSATs are focused on monitoring respiratory events or a combination of respiratory and sleep events. Among the products analyzed, 19 (32.8%) devices provided default measures of respiratory events, 23 (39.7%) devices recorded respiratory events and performed sleep analysis, four (6.9%) devices performed sleep and other analyses, one (1.7%) device performed other analyses, two (3.4%) devices were not otherwise specified, and nine (15.5%) devices measured signals only (denoted as not applicable). The number of cases with respiratory events considered to be the default measure (15 of 31, 48.4%) equaled the number of cases that exhibited both respiratory and sleep events as the default measure (15 of 31, 48.4%) when the intended use included both home and clinic/healthcare. However, the number of cases that exhibited both respiratory and sleep events as the default measure (8 of 15, 53.3%) was approximately twice as high as the number of cases that exhibited only respiratory events as the analytic metric (4 of 15, 26.7%) when the intended use was home only. Twenty-eight products (48.3%) underwent clinical trials (Y), 21 products (36.2%) had been FDA-cleared by adding or changing features unrelated to efficacy compared with a product that had already been cleared through clinical trials (N|Y), and nine products (15.5%) did not undergo clinical trials (N). Consequently, 84.5% of FDA-cleared HSAT devices had undergone clinical trials; the majority of these included parameters related to breathing events and sleep analysis. Most untested devices did not require hardware modifications or further performance evaluation (denoted as “not applicable”).

### Ensuring safety for HSAT

**Fig. 3:**
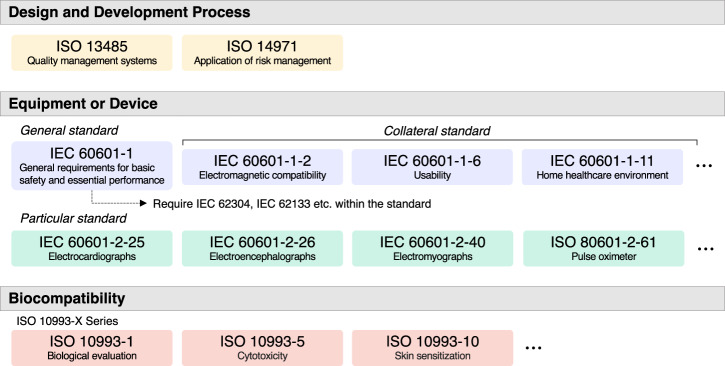
Standards used in FDA-cleared HSAT devices. FDA Food and Drug Administration, HSAT home sleep apnea testing, IEC International Electrotechnical Commission, ISO International Organization for Standardization.

Standards used in HSAT devices are structured as shown in Fig. 3. The design and development process adheres to the International Standardization Organization (ISO) 13485 and ISO 14971 standards for medical device quality management and risk management. ISO 13485 outlines fundamental requirements for a quality management system for medical devices across the entire life cycle of a product. ISO 14971 offers comprehensive guidance on the risk management process for medical devices, covering the identification, assessment, control, and monitoring of potential hazards. The application of standards for the equipment or device is in accordance with the International Electrotechnical Commission (IEC) standard 60601, a general standard specifying basic safety and essential performance requirements for medical devices. Additionally, collateral standards are mainly applied, predominantly addressing electromagnetic compatibility (IEC 60601-1-2), usability (IEC 60601-1-6), and the home healthcare environment (IEC 60601-1-11). The types of biosignals measured by HSAT devices can vary in different products, thus leading to different products adhering to various combinations of standards. This variation is product-specific; however, the most common standards applied include pulse oximeters (ISO 80601-2-61), electrocardiographs (IEC 60601-2-25), electroencephalographs (IEC 60601-2-26), and electromyographs (IEC 60601-2-40). Standards for biocompatibility are set to ensure that a material or product is safe for human use through various biological assessments, such as cytotoxicity, irritation, or intracutaneous reactivity, and is applicable when the product contains body contact elements such as straps, housings, and sensors; products meeting these standards were found to be compliant with the ISO 10993 series. We analyzed the extent to which standards were applied by category for 44 (76.9%) of the 58 total selected products and excluded 14 (23.7%) products with no specified reference standard (Table 1). The results showed that 95.5% of the reports specified standards related to electrical safety and electromagnetic compatibility (EMC), and 52.3% specified standards related to biocompatibility. The risk management category was mentioned in 45.5% of the reports. The performance and function test category, which includes particular standards for pulse oximeters, ECGs, and EEGs (among others), was mentioned in 29.5% of the reports. Additionally, the standards pertaining to usability and software validation were mentioned in 29.5% of the reports. Standards for cybersecurity, battery safety, and degree of protective packaging were cited in 20.5, 13.6, and 13.6% of reports, respectively. Related standards to quality management systems were mentioned in 6.8% of reports. Technical information reports not mandated by regulatory bodies or official standards from international standardization organizations or reports undergoing nonstandardized validation were referenced in 20.5% of reports (denoted as “others”). The majority of FDA-cleared HSAT comply with electrical safety and EMC standards, and biocompatibility was also confirmed in approximately half of the reports. Criteria such as performance and function testing, usability, software validation, and cybersecurity may be key considerations.

**Table 1:** Standards of FDA-cleared HSAT devices

Category	Standards	Percentage listed in the report (%)
Electrical safety	IEC/EN/ANSI/AAMI ES60601-1, IEC 60601-1-1, IEC 60601-1-10, IEC 60601-1-11, ANSI/AAMI EC3S, ANSI/AAMI EC57	95.5
Electromagnetic compatibility	IEC/EN 60601-1-2, IEC 60601-1-4, IEC 61000-4-2, IEC 61000-4-3, IEC 61000-4-4, IEC 61000-4-5, IEC 61000-4-6, IEC 61000-4-8, IEC 61000-4-11, CISPR 11, ANSI IEEE C63.27, EN 300 328/l 1.8.1, EN 301 489-1, EN 301 489-17, FCC Part 15B, FCC Part 2.1091, 15.247	95.5
Biocompatibility	ISO 10993, ISO 10993-1, ISO 10993-5, ISO 10993-10	52.3
Risk management	ISO 14971, FDA draft guidance	45.5
Performance and functional Tests	IEC 60601-2-25, IEC 60601-2-26, IEC 60601-2-40, ISO 80601-2, ISO 80601-2-61	29.5
Usability	IEC 60601-1-6, IEC 62366, IEC 62366-1, FDA guidance	29.5
Software validation	IEC 62304	29.5
Cyber security	ISO/IEC 27002, FDA guidance	20.5
Battery safety	IEC 62133, IEC 62133-2	13.6
Degrees of protection package	IEC 60529, IEC 60068-2-1	13.6
Quality management systems	ISO 13485	6.8
ETC	AAMI TIR 12, AAMI TIR 30, AAMI TIR 69, Implemented Not Standardized	20.5

*FDA* Food and Drug Administration, *HSAT* home sleep apnea testing, *IEC* International Electrotechnical Commission, *EN* European Norm, *ANSI* American National Standards Institute, *AAMI* The Association for the Advancement of Medical Instrumentation, *EC* ECG Committee, *CISPR* Comité International Spécial des Perturbations Radioélectriques, *IEEE* Institute of Electrical and Electronics Engineers, *FCC* Federal Communications Commission, *ISO* International Organization for Standardization, *TIR* Technical Information Report.

### Ensuring clinical efficacy

**Fig. 4:**
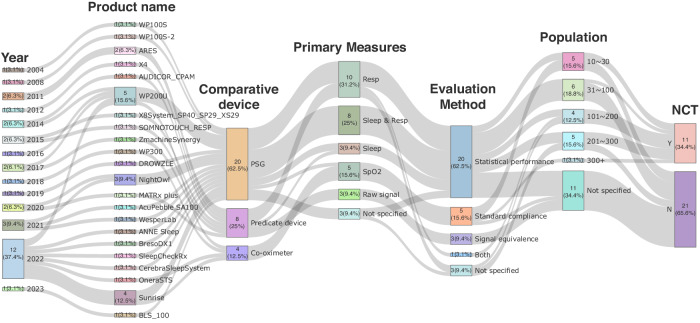
Sankey diagram for clinical trials of FDA-cleared HSAT devices. FDA Food and Drug Administration, HSAT home sleep apnea testing, NCT national clinical trial, PSG polysomnography.

Figure 4 shows a Sankey diagram of the key points of clinical effectiveness in FDA-cleared HSATs. The number of FDA-cleared products after clinical trials was usually less than three (3.1–9.4%) per year but dramatically increased to 12 (37.4%) in 2022. Particularly, WP200U (Itamar Medical Ltd., Caesarea, Israel), Sunrise (Sunrise SA, Brussels, Belgium), NightOwl (Ectosense NV, Bosbessenlaan, Belgium), and ARES (Advanced Brain Monitoring Inc., Carlsbad, USA) received five (15.6%), four (12.5%), three (9.4%), and two (6.3%) FDA clearances, respectively, indicating that multiple clinical validations have been performed for the same product. In clinical trials, PSGs, predicate devices, and CO-oximeters were employed as comparative devices for 20 (62.5%), eight (25.0%), and four (12.5%) products, respectively. Regarding primary measures, 10 (31.2%) of the products included respiratory parameters, eight (25.0%) both sleep parameters and respiratory parameters, three (9.4%) only sleep parameters, and five (15.6%) only peripheral oxygen saturation (SpO_2_). Additionally, three (9.4%) compared the similarities of the raw signals measured with existing devices, and three (9.4%) were not specified. PSG was predominantly used as a comparative device in cases in which the primary measure was “respiration only” or “sleep only,” whereas only a few devices were validated by comparison with predicate devices. All devices measuring the sleep and respiration parameters had been validated against PSG. All validations had been performed with the predicate devices in clinical trials, confirming the similarity of raw signals. Most products exclusively measuring SpO_2_ had been validated against a CO-oximeter, and only one product was validated against PSG. Some devices (9.4%) did not specify a primary measure. In performance assessments, 20 reports (62.5%) employed statistical measures like correlation, sensitivity, and specificity. Five reports (15.6%) evaluated compliance with the ISO or IEC standards, whereas three reports (9.4%) assessed signal equivalence with prior devices. One report (3.1%) utilized a combination of both methods, and three reports (9.4%) did not specify a particular evaluation method. The performance of devices related to respiratory events or sleep analysis has been predominantly assessed using statistical methods. In contrast, evaluation has exclusively been conducted based on compliance with standards when the primary measure is SpO_2_. In reports submitted for FDA clearance, five cases (15.6%) had a study population in the range of 10–30, six cases (18.8%) studied 31–100, four cases (12.5%) studied 101–200, five cases (15.6%) studied 201–300, and one case (3.1%) studied 300 or more people. The number of participants was not specified in 11 cases (34.4%). The number of participants in clinical trials was in the range of 10–300. Additionally, 11 studies (34.4%) were registered with a national clinical trial (NCT) number on ClinicalTrials.gov, whereas 21 studies (65.6%) were not. Notably, studies with a relatively large number of subjects (N > 100) were more likely to be registered.

## Discussion

We reviewed devices that have already received FDA clearance and analyzed the conditions that applied to HSATs that are currently in development for FDA clearance. We identified characteristic changes in the evolution of HSATs and provided recommendations for their development and clearance. The actual development requirements were categorized into safety and clinical effectiveness, which constitute crucial aspects of medical device evaluation. Regarding safety, adherence to international standards was identified as a key consideration. Regarding clinical effectiveness, the importance of clinical trial results, particularly those related to primary measures derived through manufacturer-specific algorithms, was emphasized. Notably, no regulatory enforcement or clear criteria for recognizing clinical trial performance were identified.

Several characteristic trends derived from FDA-cleared HSAT reports can be interpreted to originate in the following context: The number of FDA clearances for HSAT devices saw a significant spike in 2022, likely attributed to the heightened prevalence of home sleep testing referrals amid the COVID-19 pandemic, which prompted a reduction in hospital visits and an increase in social distancing^23^. However, concluding whether this marks a sustained upward trend remains premature. Continuous monitoring of the number of clearances in the coming years remains necessary to discern any enduring patterns. A notable trend is the rise in Type-3 sleep monitoring devices and a decline in Type-4 devices among FDA-cleared products. Previous reviews indicate a lack of evidence supporting the independent use of Type-4 devices, and the AASM recommends Type-3 devices for routine sleep testing^24^. Type-4 devices may simplify testing; however, their clinical validity has not been fully established. Regarding device types and usage, wearable devices continued to dominate; however, a gradual increase in the adoption of patch-type monitoring devices was observed, indicating a growing emphasis on ease of use and user experience. Furthermore, the intended use has expanded beyond home settings to encompass a full range, including healthcare/clinical applications. This transition could be facilitated by advancements in sensor and analytics technology, enhancing the necessary performance attributes of HSATs, including sensor miniaturization and increased sensitivity. Respiratory analysis emerged as the primary application of HSATs, with sleep analysis included in > 45% of FDA-cleared products. Notably, no products exclusively dedicated to sleep analysis received clearance after 2011, and those with sleep analysis tended to be combined with respiratory analysis or a pulse oximeter. This contrasts with the consistent FDA clearance of products exclusively designed for respiratory analysis, a trend likely driven by the substantial demand for respiratory analysis in diagnosing conditions like obstructive sleep apnea (OSA). The more complex measurement system required for sleep analysis, involving elements such as EEG, distinguishes it from the relatively straightforward nature of respiratory analysis.

Eleven out of 21 reports submitted to the FDA for clearance between 2003 and 2013 included standards, and the number increased to 33 out of 37 between 2014 and 2023; this can be attributed to heightened manufacturer awareness of safety and the influence of diversifying use situations and expanding functions that necessitate compliance with standards. The standards covered varied according to the type of sleep monitoring device. Type-2 and Type-3 devices are more likely to specify electrical characteristics, such as electrical safety and EMC. Interestingly, Type-2 reports are more likely to list standards for risk management and performance evaluation, whereas Type-3 reports include standards for cybersecurity and degree of protection packages that are not found in other types of reports. In contrast, Type-4 devices have a significantly higher percentage associated with the category “not specified.” Several possible reasons can explain the high number of “not specified” instances in Type-4 devices. First, four out of eight reports had been submitted a long time ago (before 2010) and do not include specifications. Additionally, the subsequent two reports focused on software, such as smartphone apps, which do not apply to the standard.

The frequency of standards listed in the report has exhibited variations over the years. Notably, usability, software validation, battery safety, and quality management are increasingly prevalent in clearance submissions among the specification categories. The rise in the frequency of usability-related specifications can be interpreted as a response to the growing significance of interface design, aimed at minimizing usage errors and enhancing patient and user safety. This trend is further underscored by the expansion of device operators from specialized technicians to the general public. Furthermore, the increased occurrence of software validation specifications likely reflects the expanding use of information technology in healthcare, emphasizing the need for robust development frameworks to ensure the safety and quality of medical device software^25^. The increase in battery safety specifications is likely a result of the growing number of portable medical devices containing built-in batteries^26^. Lastly, the heightened application of ISO 13485, an international standard related to medical device quality management system (QMS), reflects the increasing emphasis on the importance of medical device quality management. ISO 13485 has been fully adopted as a QMS standard in many countries since its revision in 2016^27^.

Respiratory devices continue to be cleared by the FDA on an annual basis. In contrast, no FDA clearances have been issued since 2011 for devices that analyze only sleep-related parameters, and we observed a growing number of FDA-cleared products that analyze both sleep and respiration. Results from clinical trials for devices analyzing respiration and those focused on sleep are reported in 17 out of 28 (60.7%) and 9 out of 28 (32.1%) reports, respectively. This proportion is significantly higher than that of clinical trials for devices without specified parameters, which are described in 4 out of 28 reports (14.3%). This suggests that validation through clinical trials is common when presenting analyses related to respiration or sleep. Regarding the analysis of specific parameters in the sleep category, seven out of nine cases focused on the sleep stage as the primary parameter. AHI emerged as the most frequently analyzed parameter (9 out of 17) in the respiratory category. However, in recent years, the analysis of parameters has expanded to include additional indices, encompassing the respiratory effort index, oxygen desaturation index, and respiration disturbance index. This reflects ongoing efforts to address the recognized limitations of AHI in determining OSA severity^28^. Smaller trials, typically involving 10–30 patients, were the predominant trend. A temporary surge in the number of patients was observed during COVID-19; however, generalizing from this data proves challenging. The overall number of NCTs was limited and caused difficulties in discerning overarching trends; nevertheless, their proportion has remained stable since their inception in 2014.

The development of HSATs is expected to continue to grow in the future. However, ensuring consistency and homogeneity in the process (from device design to validation) to ensure sufficient safety and clinical effectiveness as a medical device remains challenging. The examples of FDA clearances reveal that HSATs are at least as safe as they should be as medical devices through compliance with standards for electrical safety and other safety-related contents. However, the examples exhibited a broad variation in compliance and documentation between manufacturers and devices. As HSATs are designed for use both in and out of the hospital, adherence to relevant standards for data storage, report generation, and communication with electronic health records is crucial. Although not addressed in this study, the European Union Medical Device Regulation (MDR)—particularly articles 109 and 110 related to confidentiality and data protection—offers a framework for compliance^29^. However, these acts lack detailed implementation instructions, indicating a need for clearer regulatory guidance for manufacturers. Furthermore, the lack of an established process is true for clinical trials. Disclosures about clinical trials are becoming more specific; however, no clear criteria exist for objectively assessing whether a minimum level of clinical effectiveness has been achieved, which can lead to manufacturer-specific or product-specific variations in clinical confidence. We believe that guidances for HSAT development and licensure would aid in ensuring its minimum safety and clinical effectiveness, which could include specifications that each type of HSAT must meet, clinical trial designs, and performance evaluation criteria to demonstrate effectiveness.

Comprehensively, HSATs adhere to the conventional clearance pathway for medical devices, yet no clearance guidances exist that are uniquely tailored to HSATs. The absence of specialized guidances results in a scenario wherein, even for HSATs that have obtained clearance from the FDA, the evidence supporting their performance or clinical validity often remains nebulous. This ambiguity can engender a diminution of trust in HSATs. To rectify these issues, it is of paramount importance for regulatory agencies responsible for the licensing of medical devices to establish and enforce comprehensive standards and guidances for clinical trials, thereby ensuring the robust validation of HSAT performance. The HSAT guidances are expected to play an important role in establishing standardized procedures, ensuring quality, prioritizing patient safety, maintaining consistency of interpretation, meeting ethical considerations, and facilitating evidence-based commercialization of home sleep studies.

Some points must be considered before generalizing the results of this study. First, the objective of this research was to assess the contemporary landscape based on analyses of reports submitted for FDA clearance. It is crucial to recognize that our critical observations were confined to documents that had received FDA clearance, given that regulatory bodies, apart from the FDA, do not universally disclose their certification reports. A major limitation of this study is its exclusion of devices that have obtained the CE mark, which serves as a fundamental criterion for the approval of medical devices in numerous countries that operate outside the FDA’s jurisdiction. This omission is particularly relevant given that (since 2017) devices bearing the CE mark have been regulated based on the MDR, which has not been reflected in our analysis. This could potentially introduce a bias toward conditions approved by the FDA. Therefore, when applying these results to a global context, a degree of caution is warranted to avoid overgeneralization, as our findings may not fully encapsulate the nuances of international regulatory frameworks. Second, reports for FDA clearance are voluntarily prepared and submitted by the manufacturer. In some cases, there may be practices undertaken by the manufacturer that are not documented in these reports. Therefore, the results of this study should be interpreted as indicative trends rather than findings aiming to provide a comprehensive depiction of all actions taken by manufacturers for FDA clearance. Third, for HSATs that operate in innovative ways, the Sleep, Cardiovascular, Oximetry, Position, Effort, and Respiratory (SCOPER) categorization of HSATs based on the physiological signals measured and the methods of measurement may be more effective in structurally assessing the adequacy of device safety as well as clinical trials^30^. However, as the SCOPER categorization requires very specific information about the product (e.g., number of channels, sampling frequency), which is not available in FDA documents or manufacturer manuals and is only available in journal articles for a small number of products, this study did not include an analysis of HSATs according to the SCOPER categorization. Finally, we found missing information within the NCT documents during our efforts to summarize the clinical trial information. We have marked these data as “not specified;” however, caution should be exercised during additional interpretations.

## Methods

### Search strategy

We systematically reviewed reports published between September 1, 2003, and September 1, 2023, in the 510(k) and de novo sections of the FDA website^31,32^. The search string was determined by the following steps: 1) search of the FDA database for the FDA-cleared HSAT device names featured in the Montage^33^ to collect product codes; 2) collection of keywords from the regulation names corresponding to the collected product codes; and 3) generation of a search string by combining the collected keywords with broader sleep apnea-related keywords. The final search string used was as follows: “ventilatory effort” OR “breathing frequency” OR “sleep apnea” OR “sleep monitor” OR “sleep analysis.” Among these, “ventilatory effort recorder,” “sleep apnea,” and “monitor” were keywords gleaned from the regulation name, and keywords should be no longer than two words according to FDA website search rules.

### Eligibility criteria

The eligibility criteria for the inclusion of reports in the review included the following: 1) reports retrieved from within the FDA website and 2) products that matched sleep apnea monitoring device types 2–4, as defined by the AASM^34^. Studies were excluded from the review if 1) the description of the product code was not relevant to HSAT (e.g., broader sleep technologies, sleep services, electroencephalography devices, surgical tools); 2) the product code was a subsequent product code rather than a classification product code; 3) there was no summary; or 4) it was not relevant to HSAT; for example, failing to include the minimum number of channels and required features for each device type or devices not intended for home use.

### Data extraction

Data extraction was conducted using predefined sheets in Supplementary Tables 1 and 2. The categories were organized as follows: product name, year of FDA clearance, sensor type, sensor location, intended use, product type, number of channels, diagnostic parameters, diagnostic parameter category, sleep monitoring device type, applied standard category, and clinical trial status. Notably, sleep monitoring device types were categorized (2–4) by synthesizing the contents of other categories. Furthermore, the standards were categorized based on the criteria outlined in Table 1, and the outcome measures were classified according to the criteria specified in Table 2. In Table 2, the outcome measures are highly correlated with the physiological signals being measured. Therefore, the classification according to SCOPER^30^, the 2011 proposed criteria for classifying sleep monitoring devices based on physiological signals, is also included. Additionally, information about the products in clinical trials—including year of FDA clearance, product name, comparative device, primary measure, evaluation method, research population, and the presence of an NCT number—was documented using a separate predefined data extraction sheet (Supplementary Table 3). Clinical trial information from clinicaltrials.gov was summarized if an NCT number was provided (Supplementary Table 4).

**Table 2:** Categorization of HSAT-measured parameters

Category	SCOPER category	Parameters
Sleep analysis	Sleep	Awakening index, REM sleep, SE, SOL, Sleep stage, TST, WASO
Cardiac activity analysis	Cardiovascular	AF, HIB, HR
Oxygen saturation	Oximetry	ODI, SpO_2_
Body movement	Position	PLMS
Respiratory event	Effort, Respiratory	AHI, Apnea index, CSA, Mixed apnea, Hypopnea index, OSAI, RDI, REI, RERA, Snoring

*AF* Atrial Fibrillation, *AHI* Apnea–Hypopnea Index, *CSA* Central Sleep Apnea, *HIB* Heartbeat Irregularity Burden, *HR* Heart Rate, *ODI* Oxygen Desaturation Index, *OSAI* Obstructive Sleep Apnea Index, *PLMS* Periodic Limb Movements of Sleep, *RDI* Respiratory Disturbance Index, *REI* Respiratory Event Index, *REM Sleep* Rapid Eye Movement Sleep, *RERA* Respiratory Effort Related Arousals, *SCOPER* Sleep, Cardiovascular, Oximetry, Position, Effort, and Respiratory, *SE* Sleep Efficiency, *SOL* Sleep Onset Latency, *SpO*_*2*_ Oxygen Saturation, *TST* Total Sleep Time, *WASO* Wake After Sleep Onset.

The data extraction and organization process adhered to the following criteria: First, the unspecified characteristics of the new product were described as being the same as the previous model if a manufacturer’s product shared the same operating principle and purpose of use as its previous model but specified only minor changes in device hardware or software. We selected the highest-rated type if the report stated that a device could potentially belong to multiple types. Information extraction was conducted by two independent researchers. In cases where disagreements arose between these two authors, efforts were made to achieve consensus through direct discussion. If consensus could not be reached, the decision of another independent author (H.S.) was deemed final and accepted.

### Data analysis

We analyzed the associations between each variable to examine the relationships between variables extracted from the report for FDA clearance. Each variable was systematically compared to identify specific relationships. This matching process was applied to all pairs of variables to identify those considered important. Subsequently, we performed an in-depth analysis of the associations between these variables. We compiled a comprehensive list of all standards outlined in the report for FDA clearance in the safety analysis, which served as the primary evaluation factor for FDA clearance. The goal was to identify common elements, particularly in terms of standards such as IEC and ISO. Subsequently, we categorized these elements into three distinct categories: the product design and development process, equipment body and device, and biocompatibility. Furthermore, we specifically analyzed reports from HSAT devices that had undergone clinical trials to scrutinize efficacy requirements. The primary requirements of these clinical trials were summarized. Trend analysis was conducted using a Sankey diagram, and the frequency analysis outcomes (by category) were expressed as percentages.

### Reporting summary

Further information on research design is available in the Nature Research Reporting Summary linked to this article.

### Supplementary information

Supplementary materials

Reporting Summary

## Data Availability

All data generated or analyzed during this study are included in this published article and its supplementary information files.
