# Supplementary material for: FDA-cleared home sleep apnea testing devices
Source: NPJ Digit Med. 2024 May 13;7:123. doi: 10.1038/s41746-024-01112-w (PMC11091199; doi:10.1038/s41746-024-01112-w)
Supplement: Supplementary file 1 — Supplementary materials [file 41746_2024_1112_MOESM1_ESM.docx]

**Supplementary Information**

Supplementary Table 1. General information categories for extracting data from reports for FDA clearance

| **Variable** | **Description** |
| --- | --- |
| Year | Year of FDA clearance, ranging from 2003 to 2023 |
| Type | Device type for sleep apnea test  - Type1: full, attended PSG with 14–16 channels  - Type 2: unattended PSG with > 7 channels  - Type 3: portable device with > 4 channels  - Type 4: portable device with 1–3 channels |
| Sensor type | Type of sensor attachment: wearable or patch |
| Intended use | Environment for use: home only, both (clinical/healthcare and home) |
| Default measures | Category of primary parameters being measured: sleep, respiration, oxygen saturation, otherwise |
| Clinical trial status | Status of clinical trial report: Yes(Y), Not specified in this report but yes for its previous model’s report (N│Y), No(N) |
| Standards category | Category for listed standards: electrical safety, electromagnetic compatibility, biocompatibility, risk management, performance and functional test, usability, software validation, cyber security, battery safety, degrees of protection package, quality management systems, etc. |
| PSG: polysomnography; FDA: Food and Drug Administration; Not otherwise specified (N/S) is used when the information is listed but the exact information is not known, and not applicable (N/A) is used when the information does not need to be listed | |

Supplementary Table 2. Clinical trial-related categories for extracting data from reports for FDA clearance

| **Variable** | **Description** |
| --- | --- |
| Year | Year of FDA clearance, ranging from 2003–2023 |
| Product name | Name of product |
| Comparative device | Device category for comparative analysis in clinical trials: PSG, predicate device, CO-oximeter |
| Primary measures | Primary outcome: AHI, REI, sleep stage, etc. |
| Evaluation method | Method for evaluation of efficacy of product through clinical trial: complying standards, statistical comparison |
| Number of participants | Category of populations for clinical trial: 10–30, 31–100, 101–200, 201–300, 300+ |
| National clinical trial status | NCT registration status: Yes(Y) or No(N) |
| PSG: polysomnography; FDA: Food and Drug Administration; Not otherwise specified (N/S) is used when the information is listed but the exact information is not known, and not applicable (N/A) is used when the information does not need to be listed | |

Supplementary Table 3. Detailed information from the reports of FDA-cleared devices (N=58)

| **Product**  **name** | **FDA number** | **FDA-cleared year** | **Sensor location** | **Intended use** | **Type (HW/SW)** | **Diagnostic parameter** | **Device Type** | **Category of measures** | **Category of standards** | **Clinical**  **trial** |
| --- | --- | --- | --- | --- | --- | --- | --- | --- | --- | --- |
| WP100S | K042916 | 2004 | fingertip, wrist | home or clinic | both | RDI, REM sleep | 4 | respiratory event, sleep analysis | N/S | Y |
| APNEALINK | K061405 | 2006 | N/S | home | both | N/S | 3 | N/S | Biocomp, ElectSafe, EMC, PP, CyberSec, RM | N│Y |
| APNEALINK | K070263 | 2007 | fingertip, chest, nose | home | both | REI, oximetry | 3 | respiratory event, oxygen saturation | Biocomp, ElectSafe, EMC, PP, CyberSec, RM | N│Y |
| SOMNOSCREEN | K060708 | 2007 | N/S | home or clinic | HW | N/A | 3 | N/A | N/S | N |
| SOMNOSCREEN EEG | K071556 | 2007 | N/S | home or clinic | HW | N/A | 2 | N/A | N/S | N |
| SNAP7 | K080321 | 2008 | fingertip, chest, upper lip | home | both | AHI, RDI, REI, snoring | 3 | respiratory event | ElectSafe, EMC | N |
| WP200I | K081037 | 2008 | fingertip, wrist | home or clinic | both | RDI, REM sleep | 4 | respiratory event, sleep analysis | N/S | N│Y |
| WP200S-2 | K081982 | 2008 | fingertip, wrist | home or clinic | both | AHI, RDI, REM sleep, sleep stage | 4 | respiratory event, sleep analysis | ETC | N│Y |
| WP100S-2 | K080427 | 2008 | fingertip, wrist | home or clinic | both | AHI, RDI, REM sleep, sleep stage | 4 | respiratory event, sleep analysis | N/S | Y |
| ALICE PDX | K090484 | 2009 | fingertip, chest, nose, chin, forehead | home or clinic | HW | N/A | 2 | N/A | N/S | N |
| APNEALINK PLUS | K083575 | 2009 | fingertip, chest, nose | home | both | AHI, REI, AI, ODI, oximetry, pulse | 3 | respiratory event, oxygen saturation, cardiac activity | Biocomp, ElectSafe, EMC, PP, CyberSec, RM | N│Y |
| APNEICARE CONNECTION CENTER INTERNET ANALYSIS | K082968 | 2009 | N/A | home or clinic | SW | N/A | 2 | N/A | N/S | N |
| ACCUSOM | K110486 | 2011 | fingertip, chest, nose | home or clinic | both | OSA index, mixed apnea | 3 | respiratory event | N/S | N│Y |
| ARES | K110705 | 2011 | chest, forehead | home | both | sleep stage | 3 | sleep analysis | Biocomp, ElectSafe, EMC, ETC | Y |
| ARES | K111194 | 2011 | chest, forehead | home | both | sleep stage, apnea index | 3 | respiratory event, sleep analysis | ElectSafe, EMC | Y |
| SNAP8 | K110064 | 2011 | fingertip, chest, upper lip | home | both | N/S | 3 | Not define | EMC | N |
| WP200S-3 | K102567 | 2011 | fingertip, wrist | home or clinic | both | AHI, RDI, REM sleep, sleep stage, snoring | 3 | respiratory event, sleep analysis | ETC | N│Y |
| ARES | K112514 | 2012 | chest, forehead | home | both | AI, sleep stage | 3 | respiratory event, sleep analysis | N/S | N│Y |
| X4 | K120447 | 2012 | N/S | home or clinic | HW | N/A | 2 | N/A | Biocomp, ElectSafe, EMC, Perform | Y |
| APNEALINK PRO | K131932 | 2013 | fingertip, chest, nose | home or clinic | both | AHI, REI, AI, hypopnea index, ODI, oximetry, pulse | 3 | respiratory event, oxygen saturation, cardiac activity | Biocomp, ElectSafe, EMC, Usability, SWvalid, CyberSec, RM | N│Y |
| X4System | K130013 | 2013 | forehead, chest, chin | home or clinic | HW | N/A | 2 | N/A | N/S | N |
| AUDICOR CPAM | K131883 | 2014 | chest | home or clinic | HW, SW | AI, hypopnea index, AF | 4 | respiratory event, cardiac activity | ElectSafe, EMC | Y |
| WP200U | K133859 | 2014 | fingertip, wrist | home or clinic | both | AHI, RDI, REM sleep sleep stage, snoring, AF | 3 | respiratory event, sleep analysis, cardiac activity | ElectSafe, EMC, Perform | Y |
| ApneaLink Air | K143272 | 2015 | fingertip, chest, nose | home or clinic | both | AHI, REI, apnea index, ODI, oximetry, pulse | 3 | respiratory event, cardiac activity, oxygen saturation | Biocomp, ElectSafe, EMC | N│Y |
| SOMNOTOUCH RESP | K140861 | 2015 | fingertip, thoracic, abdomen | home or clinic | both | AHI | 2 | respiratory event | Biocomp, ElectSafe, EMC, Usability, SWvalid, RM | Y |
| X8System SP40 SP29 XS29 | K152040 | 2015 | fingertip, chest, forehead, head, abdomen, chin | home or clinic | HW | N/A | 2 | N/A | ElectSafe, BattSafe, EMC, RM, Perform | Y |
| WP200U | K153070 | 2016 | fingertip, wrist | home or clinic | both | AHI, RDI, sleep stage, snoring | 3 | respiratory event, sleep analysis | ElectSafe, EMC, Perform, ETC | Y |
| ARES | K160499 | 2017 | chest, forehead | home | both | sleep stage, oximetry | 3 | sleep analysis, oxygen saturation | Biocomp, ElectSafe, EMC, Usability | N│Y |
| MATRx plus | K163665 | 2017 | nose, mouth | home or clinic | both | ODI, oximetry, pulse | 3 | oxygen saturation, cardiac activity | ElectSafe, EMC, SWvalid | N│Y |
| WP200U | K161579 | 2017 | fingertip, chest, chest | home or clinic | both | AHI, RDI, sleep stage, snoring | 3 | respiratory event, sleep analysis | ElectSafe, EMC, ETC | Y |
| ZmachineSynergy | K172986 | 2017 | chest, nose, ear, neck | home or clinic | HW | N/A | 2 | N/A | ElectSafe, EMC | Y |
| MATRx Plus | K181996 | 2018 | nose, mouth | home or clinic | both | AHI, ODI, AI, oximetry, pulse | 3 | respiratory event, oxygen saturation, cardiac activity | ElectSafe, EMC, SWvalid | N│Y |
| WP300 | K180775 | 2018 | fingertip, chest, wrist | home or clinic | both | AHI, RDI, sleep stage, snoring | 3 | respiratory event, sleep analysis | ElectSafe, EMC, ETC | Y |
| DROWZLE | K173974 | 2019 | N/A | home or clinic | SW | OSA index | 4 | respiratory event | N/S | Y |
| MATRx Plus | K191925 | 2019 | nose, mouth | home or clinic | both | AHI, ODI, AI, oximetry, pulse | 3 | respiratory event, oxygen saturation, cardiac activity | ElectSafe, BattSafe, EMC, Usability, SWvalid, Perform | N│Y |
| WPOne | K183559 | 2019 | fingertip, chest, wrist | home or clinic | both | AHI, RDI, sleep stage, snoring | 3 | respiratory event, sleep analysis | ElectSafe, EMC, ETC | N│Y |
| MATRx plus | K200695 | 2020 | nose, mouth | home or clinic | both | AHI, ODI, AI, oximetry, pulse | 3 | respiratory event, oxygen saturation, cardiac activity | ElectSafe, BattSafe, EMC | Y |
| NightOwl | K191031 | 2020 | fingertip | home | both | AHI, TST, oximetry, pulse, REM sleep | 3 | respiratory event, sleep analysis, oxygen saturation, cardiac activity | Biocomp, ElectSafe, EMC, CyberSec, Perform | Y |
| Rubicon screening device | K200654 | 2020 | nose | home | both | Airflow event, SE, ST | 4 | respiratory event, sleep analysis | ElectSafe, EMC | N│Y |
| ApneaTrak | K192624 | 2020 | fingertip, chest, nose | home or clinic | both | AI, TST | 2 | respiratory event, sleep analysis | ElectSafe, EMC, Usability, SWvalid, Perform | N |
| SOMNOscreenPlus | K201054 | 2020 | fingertip, chest, forehead, head, abdomen, chin | home or clinic | HW | N/A | 2 | N/A | Biocomp, ElectSafe, EMC, Usability, RM, QM | N |
| AcuPebble SA100 | K210480 | 2021 | suprasternal notch | home or healthcare | both | AHI, ODI | 3 | respiratory event, oxygen saturation | Biocomp, ElectSafe, EMC, Usability, SWvalid, RM, QM | Y |
| NightOwl | K213463 | 2021 | fingertip | home | both | TST, AHI | 3 | respiratory event, sleep analysis | Biocomp, ElectSafe, EMC | Y |
| WesperLab | K203343 | 2021 | chest, abdomen | home or clinic | both | AI | 3 | respiratory event | Biocomp, ElectSafe, PP, SWvalid | Y |
| ANNE Sleep | K220095 | 2022 | fingertip, chest | home or clinic | both | AHI, oximetry, pulse | 3 | respiratory event, oxygen saturation | Biocomp, ElectSafe, EMC, Usability, SWvalid, CyberSec, Perform | Y |
| BresoDX1 | K220012 | 2022 | fingertip, suprasternal notch | home or clinic | both | AI, hypopnea index | 3 | respiratory event | Biocomp, ElectSafe, EMC, Usability, CyberSec | Y |
| NightOwl | K220028 | 2022 | fingertip | home | both | AHI, REM sleep, TST | 3 | respiratory event, sleep analysis | Biocomp, ElectSafe, EMC | Y |
| SleepCheckRx | K213360 | 2022 | N/A | home | SW | AHI | 4 | respiratory event | N/S | Y |
| WP200U | K203839 | 2022 | fingertip, chest, wrist | home or clinic | both | AHI, RDI, sleep stage, snoring, AF | 3 | respiratory event, sleep analysis, cardiac activity | ElectSafe, EMC, ETC | Y |
| WP300 | K222331 | 2022 | fingertip, chest, wrist | home or clinic | both | AHI, RDI, sleep stage, snoring, AF | 3 | sleep analysis, respiratory event, cardiac activity | N/S | N│Y |
| Sunrise | K222262 | 2022 | chin | home | both | AHI, REI, ODI, RDI, REM sleep, oximetry, pulse, TST, SOL, WASO, SE, awakening index, RERA | 3 | sleep analysis, respiratory event, oxygen saturation, cardiac activity | Biocomp, ElectSafe, EMC, CyberSec | Y |
| CerebraSleepSystem | K213007 | 2022 | fingertip, chest, forehead, head, abdomen, chin, leg | home | both | AHI, RDI, sleep stage, TST, SOL, PLMI | 2 | sleep analysis, respiratory event, Body movement | Biocomp, ElectSafe, BattSafe, EMC, SWvalid, RM, Perform, ETC | Y |
| OneraSTS | K210593 | 2022 | forehead, chest, abdomen, leg | home | both | sleep stage, oximetry | 2 | sleep analysis, oxygen saturation | ElectSafe, EMC, Usability, SWvalid, RM, Perform | Y |
| AcuPebble OX100 | K222950 | 2023 | suprasternal notch | home or healthcare | both | AHI, ODI | 3 | respiratory event, oxygen saturation | Biocomp, ElectSafe, EMC, SWvalid, RM, QM, Perform | N│Y |
| BLS 100 | K222579 | 2023 | fingertip | home or clinic | both | AHI, sleep stage | 3 | respiratory event, sleep analysis | Biocomp, ElectSafe, BattSafe, EMC, Usability, CyberSec, Perform | Y |
| WP1 | K223675 | 2023 | fingertip, wrist, chest | home or clinic | both | AHI, RDI, sleep stage, snoring | 3 | respiratory event, sleep analysis | N/S | N│Y |
| WesperLab | K221816 | 2023 | chest, abdomen | home or clinic | both | AI | 3 | respiratory event | ElectSafe, BattSafe, EMC, Usability, PP | N│Y |
| Onera STS | K223573 | 2023 | forehead, chest, abdomen, leg | home or healthcare | both | sleep stage, oximetry | 2 | sleep analysis, oxygen saturation | Biocomp, ElectSafe, EMC, Usability, SWvalid, RM, Perform | N│Y |

AF: atrial fibrillation; AHI: apnea-hypopnea index; AI: apnea index; BattSafe: battery safety; Biocomp: biocompatibility; CyberSec: cyber security; ElectSafe: electrical safety; EMC: electromagnetic compatibility; ETC: et cetera; HW: hard ware; ODI: oxygen desaturation index; PD: portable device; Perform: Performance and Functional Tests; PLMI: periodic limb movement index; PP: degrees of protection package; QM: quality management systems; RDI: respiratory disturbance index; REI: respiratory event index; REM sleep: rapid eye movement sleep; RERA: respiratory effect related arousals; RM: risk management; SE: sleep efficiency; SOL: sleep onset latency; SW: software; SWvalid: software validation; TST: total sleep time; WASO: wake after sleep onset.

N: no; N/A: not applicable; N/S: not otherwise specified; N│Y: Not specified in this report but yes for its previous model’s report; Y: yes

Supplementary Table 4: Detailed Specifications of clinical trials (N=28)

| **Product name** | **510(k) number** | **Device type** | **NCT status** | **Year** | **Country** | **Population** | **Age group** | **Primary measures** | **Comparative device** |
| --- | --- | --- | --- | --- | --- | --- | --- | --- | --- |
| AcuPebble SA100 | K210480 | 3 | Y | 2021 | UK | 150 | adults | N/S | PSG |
| ANNE Sleep | K220095 | 3 | Y | 2022 | USA | 287 | adults | AHI | PSG |
| ARES | K110705 | 3 | N | 2011 | N/S | 14 | N/S | sleep stage | Predicate device |
| ARES | K111194 | 3 | N | 2011 | N/S | N/S | N/S | respiratory effort | Predicate device |
| AUDICOR_CPAM | K131883 | 4 | N | 2014 | Taiwan | 77 | N/S | apnea index, hypopnea index, AF | Predicate device |
| BLS_100 | K222579 | 3 | Y | 2023 | USA | 100 | adults | REI, sleep stage | PSG |
| BresoDX1 | K220012 | 3 | Y | 2022 | N/S | 164 | N/S | REI, ODI, SpO_2_, pulse | PSG |
| DROWZLE | K173974 | 4 | Y | 2019 | USA | 272 | adults | AHI | PSG |
| MATRx plus | K200695 | 3 | N | 2020 | N/S | N/S | N/S | AHI | Predicate device |
| NightOwl | K191031 | 3 | Y | 2020 | Belgium | 30 | all age | SpO2 | PSG |
| NightOwl | K213463 | 3 | N | 2021 | N/S | N/S | N/S | AHI | PSG |
| NightOwl | K220028 | 3 | Y | 2022 | USA | 106 | all age | AHI, TST | PSG |
| SleepCheckRx | K213360 | 4 | N | 2022 | N/S | 228 | adults | OSA index | PSG |
| SOMNOTOUCH_RESP | K140861 | 2 | N | 2015 | N/S | N/S | N/S | AHI | Predicate device |
| WP100S | K042916 | 4 | N | 2004 | N/S | N/S | N/S | sleep stage | PSG |
| WP100S-2 | K080427 | 4 | N | 2008 | N/S | N/S | N/S | sleep stage, RDI, AHI | PSG |
| WP200U | K153070 | 3 | N | 2016 | N/S | N/S | adolescent | AHI, sleep stage | PSG |
| WP200U | K161579 | 3 | N | 2017 | N/S | N/S | N/S | sleep analysis, respiratory event | PSG |
| WP200U | K203839 | 3 | Y | 2022 | Israel | 274 | all age | N/S | PSG |
|  |  | 3 | Y | 2022 | USA, Canada, Israel | 200 | all age | N/S | PSG |
| WP200U | K133859 | 3 | Y | 2014 | USA | 500 | adults | SpO_2_ | CO-oximeter |
| WP300 | K180775 | 3 | N | 2018 | N/S | 11 | adults | SpO_2_ | CO-Oximetry |
| WesperLab | K203343 | 3 | N | 2021 | N/S | 45 | adults | AHI | PSG |
| Sunrise | K222262 | 3 | N | 2022 | Belgium | 289 | N/S | TST, AHI, ODI, RDI | PSG |
|  |  | 3 | N | 2022 | France | 31 | N/S | TST, AHI, ODI, RDI | PSG |
|  |  | 3 | N | 2022 | Belgium | 10 | N/S | body position | PSG |
|  |  | 3 | N | 2022 | N/S | N/S | N/S | SpO_2_ | CO-oximeter |
| CerebraSleepSystem | K213007 | 2 | N | 2022 | N/S | 84 | N/S | TST, AHI, RDI, sleep stage  , PLMI | PSG |
| OneraSTS | K210593 | 3 | Y | 2022 | USA | 14 | N/S | SpO_2_ | CO-oximeter |
| X8System_SP40_SP29_XS29 | K152040 | 2 | N | 2015 | N/S | N/S | N/S | raw signal | Predicate device |
| ZmachineSynergy | K172986 | 2 | N | 2017 | N/S | N/S | N/S | raw signal | Predicate device |
| X4 | K120447 | 2 | N | 2012 | N/S | N/S | N/S | raw signal | Predicate device |
| AF: atrial fibrillation; AHI: apnea-hypopnea index; ODI: oxygen desaturation index; PLMI: periodic limb movement index; PSG: polysomnography; RDI: respiratory disturbance index; REI: respiratory event index; TST: total sleep time  N/S: not otherwise specified | | | | | | | | | |
